# Health Tract, No. 299

**Published:** 1867-09

**Authors:** 


HEALTH TRACT, No. 299.

---

### Cereal Food.

THE grains proper of this country are not appreciated as they ought to be, for daily food at our tables; these are Indian corn, Wheat, Rye, Barley, and oats; they contain all the elements of nutrition necessary to the support of the human system, and if they could be used for two of the daily meals, as breakfast and supper, without anything else, there would be an incalculable advantage to the soundness of the teeth, the strength of the bones, the hardness of the muscles, the endurance of the body, and the vigor of the brain. They can be all made into bread after having been reduced to flour, but not only is this at a serious loss of nutriment, but it involves a useless waste of digestive power. Wheat bread requires three hours and a half to be digested in an ordinary stomach; boiled wheat will be digested in two hours. Boiled barley has ninety-two per cent. of nutriment; flour in the form of bread, from thirty to eighty per cent. But there is another important practical consideration, relative to children. Seventy-one parts out of a hundred of the body of the teeth are composed of lime, and of the enamel of the tooth, that which preserves it from decay, being its external coating, ninety-four per cent. is of lime. This lime comes chiefly from the bread we eat; but in converting the ordinary grain into flour, the bran, the husk of the grain, is separated from the flour, yet it is this bran which contains the lime in the largest proportions; thus, in five hundred pounds of fine flour, there are thirty pounds of bone; in five hundred pounds of the whole grain, there are eighty-five pounds of bone; and when it is considered how much teeth add to personal beauty, and how important they are to the healthful preparation of the food for the stomach, thus saving stomach labor, it is not easy to estimate properly the advantage which the whole of grain, as food, has over the flour preparations. We never become weary of bread, butter, potatoes, and some other articles, and if the cereals were well prepared, cooked thoroughly and judiciously seasoned, there is no doubt they could be made as palatable the year round as good bread

The grain should be taken whole, or broken into several pieces, covered with warm water, placed on the stove or fire, to remain there three or four hours, then boil slowly for several hours longer, with an occasional stirring, until quite soft and thick; then eat with milk or butter or syrup or salt; or if cold, slice off and fried brown. If from two to fifteen, children were compelled to make two of the three meals a day of these preparations of whole grain, or parching it brown like coffee, and eaten with boiled milk, after being itself well boiled in all cases of loose bowels, a great gain would be made in personal beauty, manly vigor, physical endurance, and mental power. — FROM THE BOSTON WATCHMAN & REFLECTOR.

